# 林普利塞治疗复发难治性自身免疫性溶血性贫血快速起效1例

**DOI:** 10.3760/cma.j.cn121090-20250812-00378

**Published:** 2026-03

**Authors:** 紫薇 刘, 苗 陈

**Affiliations:** 中国医学科学院北京协和医院血液内科，北京 100730 Department of Hematology, Peking Union Medical College Hospital and Chinese Academy of Medical Sciences, Beijing 100730, China

患者，女，54岁。2017年因面色苍白、乏力、双下肢水肿就诊外院，查血常规：HGB 53 g/L，网织红细胞比值（Ret％）28.5％。血生化：丙氨酸转氨酶（ALT）11 U/L，总胆红素（TBIL）37.7 µmol/L，直接胆红素（DBIL）6.8 µmol/L；抗核抗体（ANA）（+）均质颗粒型1∶1280，抗dsDNA抗体、抗Sm抗体、抗SSA、Coombs试验、血免疫固定电泳均阴性；抗心磷脂抗体（ACL）-IgA 10.9 U/ml（正常参考值：0～1.5 U/ml），抗β₂-GP1抗体-IgA 11.3 RU/ml（正常参考值：0～4 RU/ml）；尿常规、血清铁蛋白、维生素B_12_、叶酸正常。骨髓涂片提示增生明显活跃，红系增生显著。诊断“未分化结缔组织病，溶血性贫血”。予甲泼尼龙80 mg/d，2周后序贯为泼尼松减量维持，HGB上升，80～90 g/L，患者乏力减轻，水肿消失。2019年初再次出现乏力，伴双侧掌指关节、近端指间关节胀痛、低热，复查HGB 64 g/L，TBIL 65 µmol/L，DBIL 8.6 µmol/L，ANA（+）1∶320。采用甲泼尼龙80 mg/d及霉酚酸酯0.5 g每日2次治疗，治疗2周后HGB达80 g/L。Coombs试验、冷凝集试验、红细胞渗透脆性试验、流式阵发性睡眠性血红蛋白尿症克隆检测均未见异常。

患者2019年3月就诊于我院，查血常规：WBC 6.45×10^9^/L，HGB 76 g/L，PLT 323×10^9^/L；ANA（+）均质型1∶160，补体C3 0.704 g/L（正常参考值：0.730～1.460 g/L）；补体C4、超敏C反应蛋白、红细胞沉降率、免疫球蛋白定量、尿常规均正常，ACL、抗β_2_-GP1抗体、狼疮抗凝物、巨细胞病毒DNA及EB病毒DNA为阴性。根据2012年系统性红斑狼疮国际临床合作组（SLICC）分类标准，考虑符合系统性红斑狼疮，继发Coombs试验阴性自身免疫性溶血性贫血（AIHA）。予甲泼尼龙联合他克莫司1 mg每日2次（维持谷浓度4～10 ng/ml）治疗，HGB 92 g/L。2019年至2024年间，患者溶血反复发作（HGB波动于56～95 g/L，长期依赖输血），且直接Coombs试验于2023年转为阳性，IgG型。期间历经大剂量甲泼尼龙、霉酚酸酯、他克莫司、利妥昔单抗、硼替佐米、西罗莫司、环磷酰胺及环孢素A等多线免疫抑制剂治疗，均未能维持缓解。2025年6月18日HGB下降至54 g/L，Ret％ 23.44％，淋巴细胞绝对计数（LYM）0.53×10^9^/L；TBIL 32.5 µmol/L，DBIL 8.9 µmol/L，LDH 293 U/L，血肌酐（Cr）87 µmol/L；直接Coombs试验阳性，IgG型（+）；CD4^+^ T细胞计数162个/µl，调节T细胞计数（Treg）10个/µl；ANA（+）H 1∶80，ACL、抗β2GP1抗体均阴性，补体C3、C4，总补体活性（CH50），免疫球蛋白定量，尿常规均正常，补体及ANA滴度变化与溶血活动无明显相关性。停用以上药物，仅保留泼尼松5 mg/d。2025年6月25日开始服用林普利塞40 mg/d，同时服用阿昔洛韦、复方磺胺甲�唑预防感染。服药1周后复查HGB 90 g/L，Ret％ 9.29％，WBC 3.5×10^9^/L，ANC 2.56×10^9^/L，LYM 0.72×10^9^/L，胆红素降至正常，脱离输血；服药4周后HGB 95 g/L，Ret％ 4.87％，WBC 3.5×10^9^/L，LYM 0.93×10^9^/L；服药8周后HGB 108 g/L，Ret％ 6.32％，WBC 4.48×10^9^/L，LYM 1.07×10^9^/L。根据中国成人自身免疫性溶血性贫血诊疗指南（2023年版），疗效评估为部分缓解（PR）。监测肝肾功能正常，患者无不适症状，持续服用林普利塞40 mg/d及泼尼松5 mg/d治疗。

讨论：AIHA是自身红细胞抗体导致的溶血性贫血。选择性PI3Kδ抑制剂帕萨利司（Parsaclisib）治疗复发难治AIHA的临床试验中，75％（12/16）患者达到红系反应。林普利塞为中国原研的高选择性PI3Kδ抑制剂，已获批用于治疗复发难治滤泡性淋巴瘤。本例AIHA患者病程8年，历经多线免疫抑制剂治疗，反复复发，重度贫血，在服用林普利塞后1周获得快速反应，未出现明显不良反应及感染。

